# Genomic Distribution of Pro-Virulent *cpdB*-like Genes in Eubacteria and Comparison of the Enzyme Specificity of CpdB-like Proteins from *Salmonella enterica*, *Escherichia coli* and *Streptococcus suis*

**DOI:** 10.3390/ijms24044150

**Published:** 2023-02-19

**Authors:** João Meireles Ribeiro, José Canales, María Jesús Costas, Alicia Cabezas, Rosa María Pinto, Miguel García-Díaz, Paloma Martín-Cordero, José Carlos Cameselle

**Affiliations:** 1 Grupo de Enzimología, Departamento de Bioquímica y Biología Molecular y Genética, Facultad de Medicina y Ciencias de la Salud, Universidad de Extremadura, 06006 Badajoz, Spain; 2 Unidad de Aparato Digestivo, Hospital de Zafra, Área de Salud Llerena-Zafra, Servicio Extremeño de Salud, 06300 Zafra, Spain; 3 Servicio de Microbiología, Hospital Universitario de Badajoz, Servicio Extremeño de Salud, 06006 Badajoz, Spain

**Keywords:** eubacteria, virulence, *cpdB* gene, *sntA* gene, genomic distribution, 3′-nucleotidase, 2′,3′-cyclic nucleotide phosphodiesterase, cyclic oligoadenylate, c-di-AMP, c-di-GMP

## Abstract

The *cpdB* gene is pro-virulent in avian pathogenic *Escherichia coli* and in *Salmonella enterica*, where it encodes a periplasmic protein named CpdB. It is structurally related to cell wall-anchored proteins, CdnP and SntA, encoded by the also pro-virulent *cdnP* and *sntA* genes of *Streptococcus agalactiae* and *Streptococcus suis*, respectively. CdnP and SntA effects are due to extrabacterial hydrolysis of cyclic-di-AMP, and to complement action interference. The mechanism of CpdB pro-virulence is unknown, although the protein from non-pathogenic *E. coli* hydrolyzes cyclic dinucleotides. Considering that the pro-virulence of streptococcal CpdB-like proteins is mediated by c-di-AMP hydrolysis, *S. enterica* CpdB activity was tested as a phosphohydrolase of 3′-nucleotides, 2′,3′-cyclic mononucleotides, linear and cyclic dinucleotides, and cyclic tetra- and hexanucleotides. The results help to understand *cpdB* pro-virulence in *S. enterica* and are compared with *E. coli* CpdB and *S. suis* SntA, including the activity of the latter on cyclic-tetra- and hexanucleotides reported here for the first time. On the other hand, since CpdB-like proteins are relevant to host-pathogen interactions, the presence of *cpdB*-like genes was probed in eubacterial taxa by TblastN analysis. The non-homogeneous genomic distribution revealed taxa with *cpdB*-like genes present or absent, identifying eubacteria and plasmids where they can be relevant.

## 1. Introduction

The CpdB protein was first identified as a periplasmic protein encoded by the *cpdB* gene of *Escherichia coli* with attributed 3′-nucleotidase and 2′,3′-cyclic mononucleotide (2′,3′-cNMP) phosphodiesterase activities [1,2,3]. These activities have been observed also in other Gram-negative bacteria [4,5,6]. After the expression of *E. coli* CpdB as a recombinant protein, it was characterized as a highly efficient enzyme, also active on cyclic and linear dinucleotides (c-di-NMP and pNpN) [7,8]. In Gram-positives, two CpdB-like cell-wall-attached proteins named CdnP and SntA have been studied in *Streptococcus agalactiae* [9] and *Streptococcus suis* [10], respectively. They are structurally related to CpdB and also display activities as 3′-nucleotidases and phosphodiesterases of 2′,3′-cNMP, and linear or cyclic 3′,5′-dinucleotides.

The genes encoding CpdB-like proteins have been recognized as pro-virulent factors of several pathogens. The *cpdB* gene of avian pathogenic *E. coli* has been reported to favor the long-term colonization of chicken organs [11]. The *cpdB* gene of *Salmonella enterica* serovar Pullorum has been demonstrated to be pro-virulent in a chicken infection model. In the course of infection with either the wild-type or a *cpdB* mutant of *S. enterica*, similar numbers of bacteria were found in internal organs for up to 5–6 days post infection. Interestingly, the *cpdB* mutant was cleared relatively quickly, while the wild type persisted in high numbers for up to 16 days [12]. Similar attenuation of virulence occurs in *cpdB* mutants of *S. enterica* serovar Enteritidis [13]. The *sntA* gene of *S. suis* has been also reported to be pro-virulent in pigs [14,15] and the *cdnP* gene of *S. agalactiae* in mice [9]. In contrast to all these studies, no evidence was obtained for pro-virulence of the *cpdB* gene of *Yersinia enterocolítica* in an oral and intravenous mouse infection model [4]. The *cpdB* gene of the plant pathogen *Pectobacterium atrosepticum* offers a different point of view, since gene mutations make the pathogen resistant to potassium tetraborate tetrahydrate used as disinfectant [16].

The pro-virulent character of the *cdnP* gene of *S. agalactiae* is related to the mitigation of the type-I interferon response of infected mice, driven by the enzymatic hydrolysis of extracellular c-di-AMP by the *cdnP*-encoded protein CdnP [9]. During intracellular infections, this cyclic dinucleotide is released by the microbe in the host cytosol, where it acts as a pathogen-associated molecular pattern (PAMP) which activates immune response by interaction with the stimulator of interferon genes STING [9,17,18,19,20,21,22]. The hydrolysis of extracellular c-di-AMP by cell-wall-bound CdnP mitigates the interferon response of the host, and thus explains the pro-virulent character of the *cdnP* gene.

The pro-virulent *sntA* gene of *S. suis* is overexpressed under iron starvation [23], and it is a heme-binding protein that favors iron acquisition from infected host reservoirs, also inhibiting host antioxidant protein AOP2 [15]. Moreover, it also interferes with the complement system [14], and its protein product SntA, like *S. agalactiae* CdnP, catalyzes the hydrolysis of c-di-AMP rather efficiently [9,10]. These characteristics can explain the pro-virulence of *sntA*.

*S. enterica* is a facultative intracellular pathogen that can invade phagocytic and non-phagocytic cells of many organisms, including humans and birds [24,25,26,27,28,29], and, like *E. coli*, it contains periplasmic CpdB [30,31]. The mechanism of *cpdB* pro-virulence remains unknown other than its possible action through activation of the expression of other genes [12], or a hypothetical role in iron acquisition similar to that of *S. suis* SntA [15], taking into account that iron uptake is required for the persistence of *S. enterica* within vacuoles of fibroblasts [32]. The intermediation of extracellular c-di-AMP removal to explain *cpdB* pro-virulence is unlikely as, while there is some evidence for its presence and role in Gram-negative bacteria, this is rather limited [33,34,35,36]. However, *S. enterica*, like most Gram-negatives, produces the cyclic dinucleotide c-di-GMP. The role of c-di-GMP signaling in *S. enterica* has been recently reviewed [37], and different effects have been put forward for the cytoplasmic and the extracytoplasmic dinucleotide [29]. Cytoplasmic c-di-GMP in *S. enterica* controls the transition between biofilm and virulence, and modulates virulence phenotypes: it is a regulator of the sessility versus motility transition, with high levels favoring biofilm formation and inhibiting, for instance, the invasiveness of intestinal epithelial cells [29,37,38,39,40]. It is known that STING-dependent type-I interferon response of infected host cells can be evoked also by c-di-GMP, including infections by *S. enterica* [18,29,41,42]. For instance, in dendritic cells, c-di-GMP triggers innate immunity mediated by STING and induces T_H_17 cells [29,42]. 

**Scheme 1:**
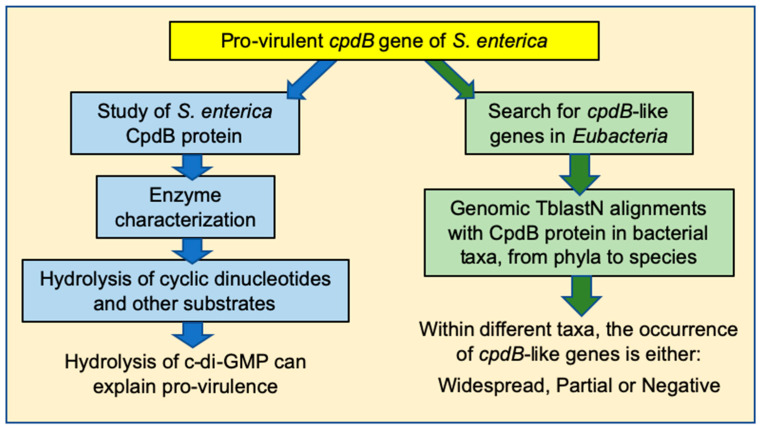
Questions addressed in this study and summary of conclusions.

In this study, based on what is known about *cpdB*-like bacterial genes and their encoded proteins, we posed two questions (see Scheme 1). Since the hypothetical hydrolytic activity of *S. enterica* CpdB towards cyclic dinucleotides could be relevant to host-pathogen interaction, an extensive study of its so-far unknown enzyme specificity was performed using a recombinant enzyme cloned from serovar Typhimurium genomic DNA. Among other things, we concluded that its detected activity on extracellular c-di-GMP could be the basis for the pro-virulent character of the *cpdB* gene of this species. However, intrigued by a casual observation that 40% of the sequenced genomes of *S. enterica* serovar Typhimurium do not contain a *cpdB* gene, we also analyzed the genomic distribution of *cpdB*-like genes in eubacteria, to explore the extent to which these genes could participate in host-pathogen interactions in different eubacterial taxa. It was concluded that *cpdB*-like genes are not ubiquitous among bacterial taxa and that, within many taxa where they occur, their distribution is not homogeneous, even at the level of species in many cases. This opens a new global perspective on the role of these genes.

## 2. Results and Discussion

### 2.1. Enzyme Characterization of S. enterica CpdB Protein

This CpdB protein, devoid of its signal sequence, was overexpressed in BL21 cells from plasmid pGEX-6P-3-S.enter_CpdB which encodes a fusion protein GST-CpdB. The recombinant enzyme present in cell lysate supernatants was purified by affinity to GSH-Sepharose and recovered free of the GST tag by specific proteolysis with PreScission protease. An extensive enzymatic characterization was performed using 3′-nucleotides, 2′,3′-cNMP, linear dinucleotides, cyclic di-, tetra- and hexanucleotides, among other substrates. The cyclic oligoadenylates c-tetra-AMP and c-hexa-AMP are second messengers produced by type III CRISPR-Cas systems [43], and there is little data about phosphodiesterases hydrolyzing them other than the so-called ring nucleases [44,45,46]. So far, c-tetra-AMP and c-hexa-AMP had not been tested as substrates of CpdB-like enzymes. 

With all the substrates of CpdB, except 2′,3′-cGAMP, saturation kinetics were studied by assaying initial rates of phosphohydrolysis at different substrate concentrations. Estimations of *k*_cat_, *K*_M_ and catalytic efficiency (*k*_cat_/*K*_M_) were obtained by non-linear regression of the Michaelis–Menten equation to the experimental data. In the case of 2′,3′-cGAMP, the catalytic efficiency was estimated from initial-rate assays directly proportional to substrate concentration. The results are shown in Table 1 in order of decreasing catalytic efficiencies.

The catalytic efficiencies for the substrates tested ranged from very high (>10^7^ M^−1^s^−1^; near the diffusion rate limit) for 3′-nucleotides, 2′,3′-cNMP and the linear dinucleotide pApA, to low values (<10^3^ M^−1^s^−1^) for c-tetra-AMP, c-hexa-AMP, 3′,5′-cAMP, NDP-hexoses, 2′,3′-cGAMP, 5′-AMP and 2′-AMP. Actually, with the two latter compounds no activity was detected, highlighting the strict specificity of the enzyme for 3′-nucleotides. Between the two extremes of catalytic efficiency, there are twelve intermediate substrates with catalytic efficiencies ranging 10^6^–10^4^ M^−1^s^−1^. Among them, pGpG, 3′,3′-cGAMP, c-di-AMP and c-di-GMP are relevant, together with pApA, to the role of cyclic dinucleotides as possible intermediates in the interferon response of the infected host. Although they are clearly worse substrates than 3′-nucleotides and 2′,3′-cNMP, they cannot be disregarded because catalytic efficiencies of 10^6^–10^4^ M^−1^s^−1^ are around the average value of catalytic efficiencies in the enzyme universe (≈10^5^ M^−1^s^−1^) [47]. 

Taking into account the periplasmic location of CpdB, one would expect that it targets extracytoplasmic c-di-GMP. In this context, the hydrolysis of c-di-GMP by the periplasmic CpdB of *S. enterica*, followed by the degradation of the pGpG product by the same enzyme, could explain the pro-virulence of the *cpdB* gene [12]. Removal of the secreted dinucleotide would hinder host immune response, a defense strategy similar to that followed by streptococci through the hydrolysis of bacterial secreted c-di-AMP to diminish the innate response of the infected host cells [9,10].

Another interesting aspect of CpdB is related to its high activity towards 2′,3′-cNMP. These compounds are formed by RNase I, an enzyme present in bacterial cytosol and periplasmic space [48,49,50,51]. Therefore, at least in the latter case, 2′,3′-cNMP formed by RNase I would be hydrolyzed by periplasmic CpdB. Recently, 2′,3′-cNMP have been proposed as a novel class of bacterial signals [50,51,52,53]. In *E. coli*, they have clear physiological effects on gene expression, flagellar motility, biofilm formation and acid tolerance. In *S. enterica*, despite the evolutionary closeness with *E. coli*, the response to 2′,3′-cNMP is quite different. To begin with, out of the many genes that are dysregulated upon 2′,3′-cNMP depletion, only two of them show consistent changes in both species. In general, it can be said that there is little overlap in the respective cellular responses [51]. Anyhow, the possible physiological impacts of extracytoplasmic 2′,3′-cNMP, and of their hydrolysis by periplasmic CpdB are unknown.

### 2.2. Comparisons of CpdB-like Enzyme Specificities of Different Bacteria

**Figure 1:**
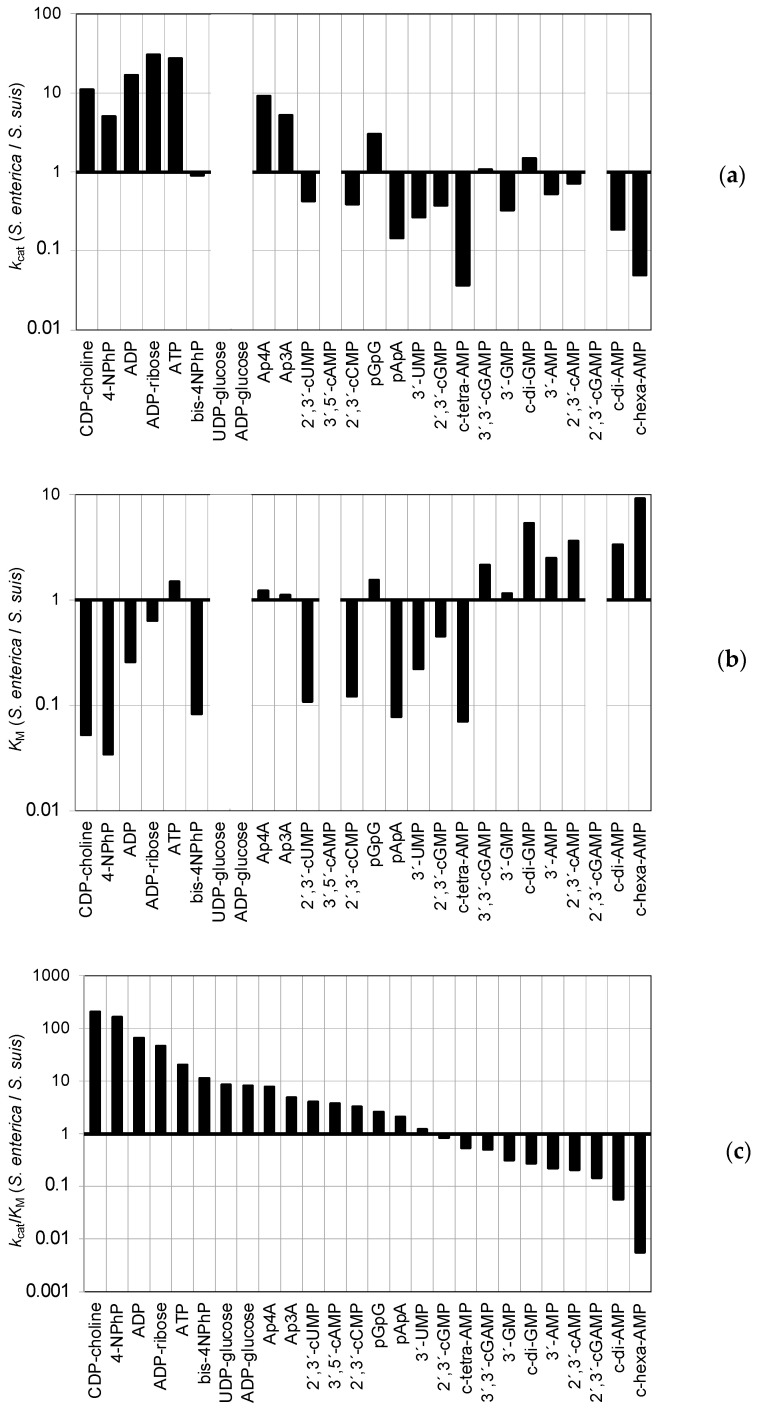
Comparison of kinetic parameters of *S. enterica* CpdB versus *S. suis* SntA for different substrates. *S. enterica* data are taken from Table 1, while those for *S. suis* were from previous work [10], except for c-tetra-AMP and c-hexa-AMP (Table 1). The bars represent parameter ratios in logarithmic scale: (**a**) ratios of *k*_cat_ values; (**b**) ratios of *K*_M_ values; (**c**) ratios of *k*_cat_*/K*_M_ values. Blank columns in panels (**a**,**b**) indicate absence of the corresponding parameter (*k*_cat_ or *K*_M_) for *S. enterica* and/or *S. suis* SntA. In the three panels, the substrates are ordered from higher to lower ratios of catalytic efficiency (*k*_cat_*/K*_M_).

**Figure 2:**
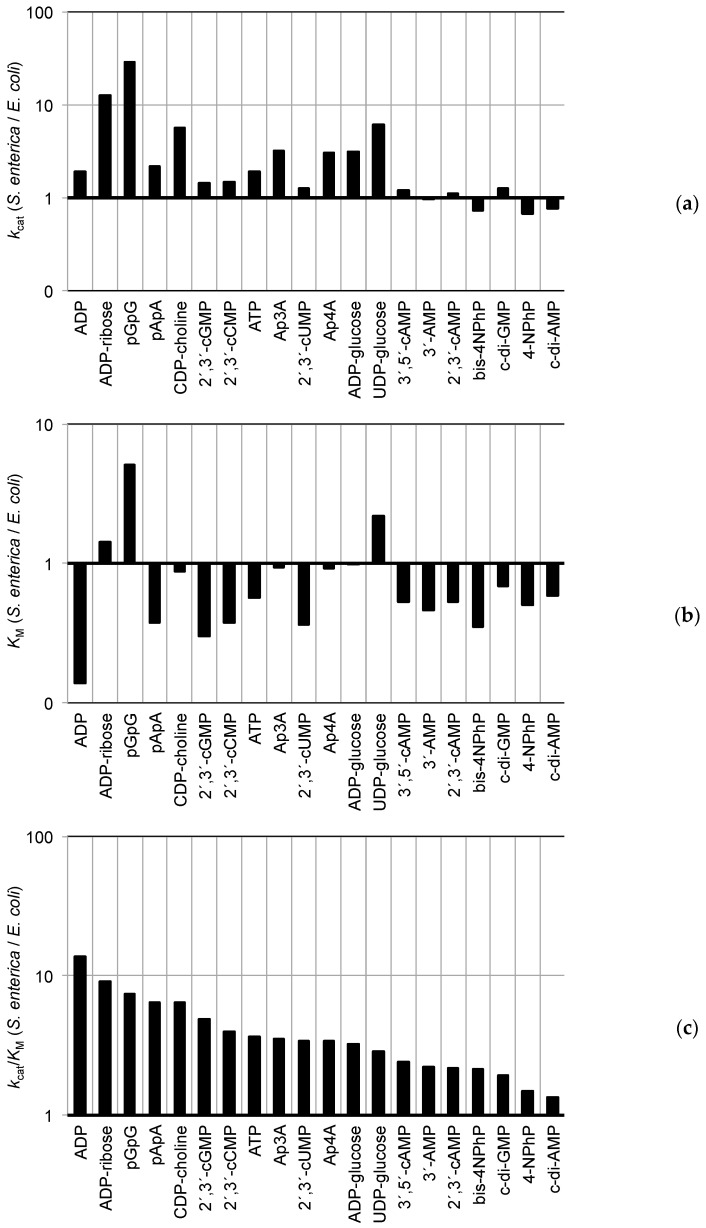
Comparison of kinetic parameters of *S. enterica* CpdB versus *E. coli* CpdB for different substrates. *S. enterica* data are taken from Table 1, while those for *E. coli* were from previous work [7]. The bars represent parameter ratios in logarithmic scale: (**a**) ratios of *k*_cat_ values; (**b**) ratios of *K*_M_ values; (**c**) ratios of *k*_cat_*/K*_M_ values. In the three panels, the substrates are ordered from higher to lower ratios of catalytic efficiency.

Besides the Table 1 data, there are two published reports of detailed substrate specificity of CpdB-like enzymes with kinetic parameters, one for *E. coli* CpdB [7] and the other for *S. suis* SntA [10]. Figure 1 presents the comparison of *S. enterica* CpdB with *S. suis* SntA, while *S. enterica* CpdB is compared to *E. coli* CpdB in Figure 2. A direct comparison between *S. suis* SntA and *E. coli* CpdB can be found elsewhere [10]. 

The comparison of *S. enterica* CpdB with *S. suis* SntA in terms of *k*_cat_/*K*_M_ (Figure 1c) revealed two substrate groups depending on the ratio of catalytic efficiencies between both enzymes being higher or lower than unity. The enzyme from *S. enterica* was less efficient than that from *S. suis* for 10 substrates, including cyclic oligonucleotides, particularly for c-hexa-AMP and c-di-AMP, and (much) less markedly for 2′,3′-cGAMP, c-di-GMP, 3′,3′-cGAMP and c-tetra-AMP. In all these cases, the lesser efficiency of *S. enterica* CpdB was generally related to higher *K*_M_ values (with the exception of c-tetra-AMP; Figure 1b) and lower *k*_cat_ values (except for c-di-GMP and 3′,3′-cGAMP; Figure 1a). On the other hand, for the other 16 substrates, the enzyme from *S. enterica* was more efficient than that from *S. suis* (Figure 1c) generally related to lower *K*_M_ (Figure 1b) and higher *k*_cat_ values (Figure 1a). 

The results of the above comparison are similar to those obtained when *S. suis* SntA is compared to *E. coli* CpdB [10], although in this case less substrates were available. The differences of SntA versus *E. coli* CpdB, were more marked than versus *S. enterica* CpdB. This reflects better in the direct comparison between both CpdB enzymes (Figure 2), where all the substrates that could be compared were more efficiently hydrolyzed by the enzyme from *S. enterica* (Figure 2c), particularly so with the linear dinucleotides, reflecting higher *k*_cat_ and lower *K*_M_ values with a few exceptions (Figure 2a,b). 

### 2.3. Structural Comparison of CpdB-like Proteins of Different Bacteria

**Figure 3:**
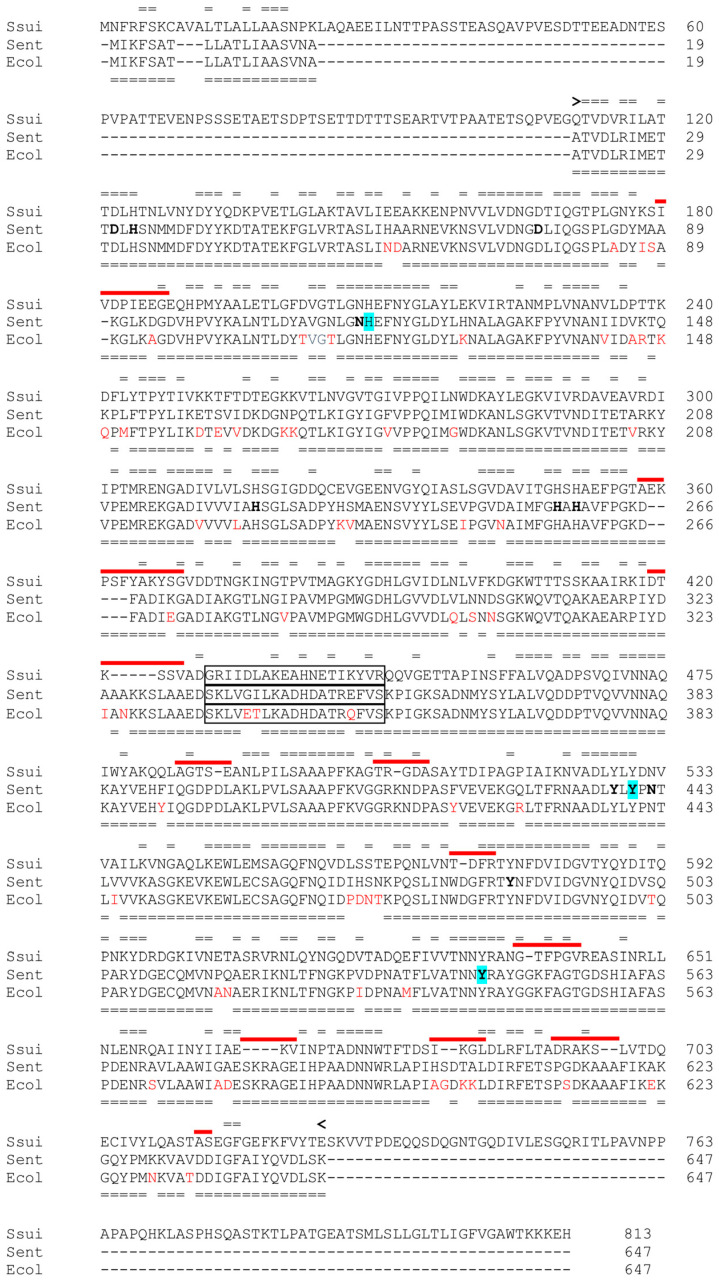
Sequence alignment of the proteins used in this study. The alignment was prepared with Clustal Omega online (https://www.ebi.ac.uk/Tools/msa/clustalo/; accessed 5 February 2023) with a few manual edits. The sequences correspond to NCBI Protein accessions: Ssui, AYV64543; Sent, P26265; Ecol, AKS04560 with the addition of the signal sequence. The symbol = above Ssui sequence and below Ecol one indicates identity with the Sent sequence. The recombinant proteins studied start and finish in the amino acids marked with **arrowheads**. The sequences within **boxes** correspond to the interdomain linkers between the N-terminal “metallophos” and the C-terminal “5_nucleotid_C” domains (see the structures shown in Figure 4). **Bold-type amino acids** in the Sent sequence are either those coordinated (in the metallophos domain) with the metal ions or located (in the 5_nucleotid_C domain) at ≤4 angstrom of a 3′-AMP substrate modeled in the active site of *E. coli* CpdB. **Shadowed in blue** are His117, which has a catalytic role in enzymes of the metallophosphatase family, Tyr440 and Tyr544, which form a sandwich with the nitrogen base of substrates like 3′-AMP [8,10]. **Red lines** above the Ssui sequence, mark the most significant differences found with respect to the Sent sequence, most of them related to alignment gaps in either sequence. **Amino acids in red type** in the Ecol sequence mark the differences with respect to the Sent sequence. All these differences are highlighted in red in the structures shown in Figure 4a,c.

The specificity differences among the three CpdB-like enzymes studied should be the consequence of sequential/structural differences among the proteins. The protein alignment of Figure 3 displays separately the differences between *S. suis* Snta and *S. enterica* CpdB (above the alignment), and those between *E.coli* CpdB and *S. enterica* CpdB (below the alignment). There are many differences between the sequences of *S. suis* SntA and *S. enterica* CpdB. The former is 813 amino acids long, and in the alignment only 283 of them are identical. Within the parts that align with *S. enterica* CpdB, there are several gaps either in SntA or CpdB. The amino acid sequences of the CpdB proteins from *S. enterica* and *E. coli* are 90.3% identical. Both proteins are 647 amino acids long, and 584 of them are identical in the alignment. They align without any gap. The differences (Figure 3) should be responsible for the different specificity of SntA versus CpdB (Figure 1c), and for the higher efficiency of *S. enterica* CpdB compared to *E. coli* CpdB (Figure 2c).

Currently, there are no crystal structures available for any of the three proteins considered, and within the AlphaFold Protein Structure Database [54,55] there is a model only for *S. enterica* CpdB (UniProt ID P26265; AF-P26265-F1-model_v4.pdb). So, to evaluate possible structural differences among the three proteins, we used homology models of *E. coli* CpdB and *S. suis* SntA prepared using the AlphaFold structure of *S. enterica* CpdB as the template. The homology models were obtained in the Phyre2 server [56]. 

**Scheme 2:**
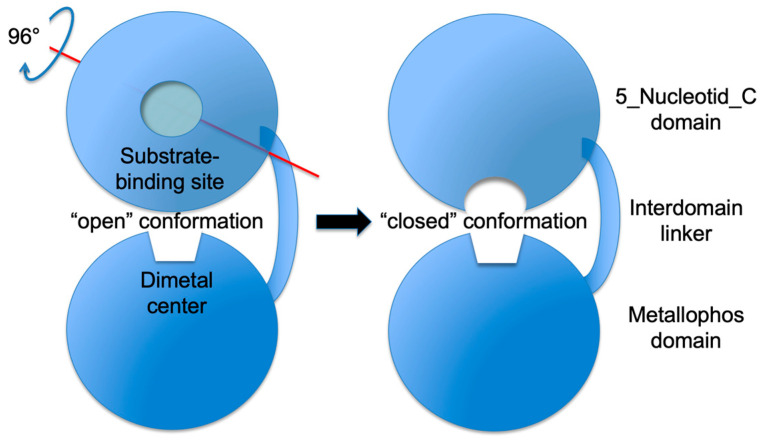
Rotation of the 5_nucleotid_C domain of UshA-like and CpdB-like metallophosphatases during the catalytic cycle.

**Figure 4:**
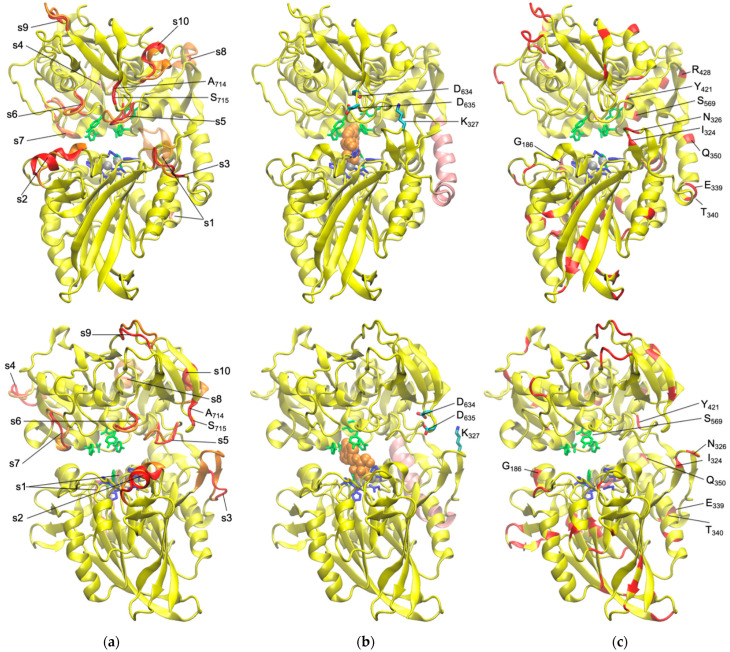
Molecular models of the CpdB-like proteins of (**a**) *S. suis,* (**b**) *S. enterica* and (**c**) *E. coli*. The three structures correspond to the parts of the proteins that were used for enzyme characterization (as indicated in Figure 3). They contain the 5_nucleotid_C domain, the interdomain linker (colored **pink** in panel (**b**)) and the metallophos domain. The upper and lower panels show the same structures with a 50° rotation. The figure shows (panel (**b**)) a 3′-AMP molecule in the active site of *S. enterica* CpdB (taken from [8]; it fits equally well in the other proteins, not shown). The dimetallic centers of the three proteins are shown (**grey** spheres). The amino acid side chains configuring the substrate binding center in the 5_nucleotid_ C domain (colored in **green**), and those coordinated to the metal ions in the metallophos domain (colored in **blue**) are all sequentially and spatially conserved in the three proteins. In the SntA structure (**a**), parts colored in **red (except A_714_–S_715_**) are those showing differences of structure with respect to CpdB (marked also in Figure 3), and those colored in **orange** depict the parts of CpdB which are substituted in SntA. In the *E. coli* CpdB structure (**c**), parts colored in **red** indicate differences of sequence with respect to *S. enterica* CpdB (marked also in Figure 3). For comments on s1–s10 and other labels, see the text of Section 2.3.

To analyze how the differences among the three proteins can have some bearing on their specificity and catalytic efficiency, it is necessary to consider the dynamic events occurring during the catalytic cycle of the metallophosphatases that contain a 5_nucleotid_C domain (Scheme 2). This is inferred from detailed studies of the 5′-nucleotidase UshA [57,58,59,60,61,62] and recently it has been extrapolated to CpdB [8]. The 5_Nucleotid_C domain contains the substrate-binding pocket, which in the “open” conformation faces the medium. After substrate binding, this domain undergoes a 96° rotation towards the “closed” conformation, bringing the scissible linkage of the substrate to the catalytic dimetallic site of the metallophos domain where phosphohydrolysis takes place. This is the conformation shown in the models of Figure 4.

The differences of sequence between *S. suis* SntA and *S. enterica* CpdB are too many to warrant a systematic analysis of all of them (there are 364 different amino acids within the aligned regions in Figure 3). Therefore, attention was centered on the gaps arising in the alignment: 17 gaps in the SntA sequence and 6 gaps in the CpdB one. They are marked by upper red lines in the SntA sequence (Figure 3) and colored in red in the 3D model (Figure 4a; s1–s10). Related to these gaps, the SntA model presents structural variations with respect to CpdB, as can be confirmed by careful comparison of Figure 4a with Figure 4b. This is underscored in Figure 4a by representing colored in orange the parts of CpdB that do not overlap with SntA. 

Most of the structural differences between SntA and the CpdB proteins are located in the 5_Nucleotid_C domain (s4–s10; Figure 4a), which is responsible for substrate binding in the open conformation (not shown), and undergoes the large rotation needed to bring the substrate to the catalytic site (Scheme 2). Several of the structural differences occur in regions near the active site in the closed conformation (s2, s5, s6 and s7), or near the region where twisting occurs during the rotation (s3, s5, s10). The most conspicuous difference is the one marked as s3, which affects amino acids 419–424 of *S. suis* SntA, that in *S. enterica* and *E. coli* CpdB proteins are substituted by amino acids 322–332 which include two lysine residues (Lys_327_ and Lys_328_) absent in SntA. In CpdB proteins, this structural variation is associated with the presence of two aspartates (Asp_634_ and Asp_636_) which are also different in SntA (Ala_714_ and Ser_715_). As can be seen in Figure 4b, Lys_327_ (in the metallophos domain) and the two aspartates (in the 5_Nucleotid_C domain) may establish an electrostatic interaction during rotation of the latter domain. This could retard the full closing of the active site of the CpdB proteins and at least partly explain some kinetic differences of efficiency (Figure 1). This analysis is complicated by the variety of substrates hydrolyzed by the enzymes, and by the possibility that the “closed” conformation is not the same with substrates of different sizes, e.g., 3′-nucleotides and cyclic dinucleotides.

Despite the 63 non-identical amino acids in the sequences of *S. enterica* and *E. coli* proteins, their 3D structures were practically undistinguishable in the overlapped models (not shown but compare Figure 4b with Figure 4c). Therefore, we centered our analysis on the differential sequences, which are highlighted in red both in the *E. coli* CpdB sequence (Figure 3) and the 3D model (Figure 4c). None of these variations appears close enough to the active site in the “closed” conformation to explain the higher efficiency shown by *S. enterica* CpdB (Figure 2c). However, one of the sequence differences (marked as Q_350_ in Figure 4c) is located in the region of the interdomain linker that twists during the large rotation suffered by the 5_Nucleotid_C domain to bring the substrate towards the catalytic site in the metallophos domain (see Scheme 2). The difference is a substitution of Gln_350_ in *E. coli* CpdB by Glu_350_ in *S. enterica* CpdB. It is conceivable that the negative charge favors the rotation and makes it occur more quickly. This would justify the larger *k*_cat_ values observed with many substrates, but it explains neither why this does not occur with all, nor the differences of *K*_M_ (Figure 2a,b). Similar reasoning can be applied to other differences near the Q_350_ mark in Figure 4c: I_324_, N_326_, E_339_, T_340_, Y_421_, R_428_ and S_569_, since they are located near the region twisted during the rotation of the 5_Nucleotide_C domain, and also interesting is G_186_, not far from the space occupied by substrates in the closed conformation. In *E. coli* CpdB, they represent significant substitutions with respect to *S. enterica* CpdB: Gly186Ile, Ile324Ala, Asn326Ala, Glu339Gly, Thr340Ile, Tyr421Phe, Arg428Gln and Ser569Ala. All of these substitutions imply differences of charge, polarity, hydrophobicity and/or size in the side chain at those positions. 

Altogether, the structural dataset provided by this study paves the way for future studies of mutagenesis to elucidate the molecular basis of the differential specificity and catalytic efficiency of the three CpdB-like proteins compared. 

### 2.4. Genomic Distribution of cpdB-like Genes in Eubacteria

To perform a systematic study of this distribution, the strategy explained in Materials and Methods Section 3.4 was applied to the *Bacteria* taxa of the NCBI Taxonomy browser [63] at different levels (Table 2, Table 3, Table 4, Table 5 and Table 6). TblastN analyses [64,65] were run using *S. enterica* CpdB (accession number P26265) as the query, with the score and query coverage limits indicated. A score limit of 150 was chosen taking into account the occurrence of CpdB-like homologs named 5′-nucleotidase/UDP-sugar hydrolase (UshA) [66,67], with a two-domain structure similar to CpdB. In BlastP comparisons, most UshA proteins align with P26265 with scores < 130 (as compared to scores > 1000 for alignments between CpdB proteins from different bacteria). Nevertheless, the limit of score 150 is somewhat arbitrary, as one cannot totally rule out that some true *cpdB* relatives align with P26265 with lower scores, while choosing a lower limit to avoid this would count some *ushA* genes as *cpdB*-like. The borderline hits in every *Bacteria* phylum (Table 2); when tested by BlastP, it showed a (much) better alignment score with CpdB than with UshA. In a few cases that this was not so, the affected hits were removed (see footnotes 4 and 3 of Table 2 and Table 3, respectively). The limit of 70% query coverage was chosen to ensure that the two domains typical of CpdB are covered by the alignment. In principle, the search was performed among genome “sequences from type material” [68], but in some cases this restriction was removed (see below).

Another point one should be aware of is that some organisms rather than, or in addition to having separate proteins CpdB and UshA, may express a natural fusion of both, as the result of two-gene fusion [69]. Such a protein was experimentally observed and characterized in *Bacillus subtilis* [70], and it is detected mainly in sequenced genomes of phylum *Firmicutes* (classes *Bacilli* and *Clostridia*). Of course, the fused genes were counted as *cpdB*-like, since P26265 aligns well with their *cpdB* moiety, and no attempt to correct this was performed. Among other things, the CpdB-UshA natural fusions may be enzymatically active [70].

Following the described search strategy and limits, out of 83,531 sequences of complete genomes of *Bacteria* (NCBI:txid2), 1772 gave significant TblastN alignments with *S. enterica* CpdB, and 984 aligned with score > 150 and query coverage > 70%. In contrast, the superkingdom *Archaea* (NCBI:txid2157) gave no significant alignments with the same limits. In Table 2, Table 3, Table 4, Table 5 and Table 6, the near one thousand *cpdB*-like genes found in *Bacteria* are shown distributed among taxonomical groups according to different levels of classification. Results obtained at the level of phylum or groups of phyla are shown in Table 2, where all the well-established phyla are included except those for which, at the time of running the final search (15 December 2022), sequenced genomes of type material were not available. Further exploration was run at the level of class, only within the phyla *Proteobacteria* and *Firmicutes* (Table 3). Thereafter, results at the level of order were obtained only for those belonging to classes *Gammaproteobacteria* and *Bacilli* (Table 4), and results at the level of family only for those belonging to orders *Enterobacterales* and *Lactobacillales* (Table 5). Finally, an extensive selection of specific examples of pathogens of clinical interest is included in Table 6. Interestingly, the genomic distribution of *cpdB*-like genes among the genomes of *Bacteria* was not homogeneous, as indicated in Table 2, Table 3, Table 4, Table 5 and Table 6 by qualification keys “N” (Negative), “Nm” (Negative, mainly), “P” (Partial), “Wm” (Widespread, mainly) and “W” (Widespread). 

Let us consider first the results obtained at the level of phyla (Table 2). The presence of *cpdB*-like genes was clear in *Proteobacteria*, *Firmicutes*, *Deinococcus-Thermus*, *Spirochaetes*, *Thermotogae, Actinobacteria* and the *FCB* group of phyla. In none of them the presence was widespread, only partial, meaning that, out of the tens to hundreds of sequenced genomes of type material for each of those phyla, between 11% and 51% gave hits indicative of *cpdB*-like genes. A wide range of scores was obtained, from near the limit of 150 to high values, which were higher in *Proteobacteria* than in the other cases (an expected result as the query is a protein from a *Proteobacteria* species; see below). In addition, the phyla *Coprothermobacterota* and *Calditrichaeota*, with a single sequenced genome each, contained a low-score hit. The rest of the phyla were either mainly negative, giving 1–2 hits with low scores in 5–221 sequenced genomes, or fully negative in 1–51 sequenced genomes. For all the phyla that gave only 0–2 hits in the available sequenced genomes of type material, the search was extended to additional genomes sequenced by removing the limit to type material (data also shown in Table 2). This revealed a small number of additional hits that did not modify the qualification key of the genomic distribution for any phylum.

In summary, out of the 27 phyla or groups of phyla with complete genomes available, *cpdB*-like genes are absent or near absent in 18, and present in the other 9 phyla. In the latter, the distribution is partial not homogeneous, with some genomes containing a *cpdB*-like gene and others not, except for two phyla with only one genome sequenced.

Further exploration of *cpdB*-like genes at levels lower than phylum was centered on *Proteobacteria* and *Firmicutes*, where there are many type-material genomes sequenced that gave hits in 30% and 39% of cases, respectively, with many high scores. There were 139 hits with score > 1000 in *Proteobacteria*, and 72 hits with scores > 500 in *Firmicutes*. The difference depends on the sequential differences between genes coding for enzymes either periplasmic (such as *S. enterica* CpdB) or cell wall-bound (such as *S. suis* SntA). When the TblastN was repeated using SntA sequence (accession AYV64543) as the query, the scores were higher for *Firmicutes* than for *Proteobacteria*. It may be remarked that the CpdB-like enzymes compared in Section 2.1 and 2.2 come either from *Proteobacteria Enterobacteriaceae* fam. (*S. enterica* and *E. coli*), or from *Firmicutes Streptococcaceae* fam. (*S. suis*). 

In Table 3, phyla *Proteobacteria* and *Firmicutes* are subdivided into classes that also showed a non-ubiquitous and non-homogeneous distribution of *cpdB*-like genes. In *Proteobacteria*, 5–42% of the type-material genomes of *Gammaproteobacteria*, *Betaproteobacteria*, *Alphaproteobacteria* and *Delta/epsilon subdivisions* gave hits with high scores. In *Firmicutes*, *Bacilli*, and *Clostridia* gave hits in 22% and 51% of the genomes, respectively. The other *Proteobacteria* and *Firmicutes* classes were mainly negative or just negative, except *Erysipelotrichia*, with a partial distribution of *cpdB*-like genes with very low scores, and *Limnochordia*, with a moderately high score in a single genome sequenced.

In Table 4, the orders pertaining to classes *Gammaproteobacteria* and *Bacilli* were analyzed. Here, for the first time in the course of the TblastN analysis, appeared a taxonomical level with 100% of the type-material genomes with hits (except some taxonomical levels with a single genome sequenced), namely the order *Pasteurellales*. In this case, repetition of the TblastN without the limit “sequences from type material” gave 86% of the total genomes with hits (460/532). In addition, the order *Enterobacterales* gave hits in 92% of the type-material genomes, while *Moraxellales*, *Vibrionales*, *Alteromonadales*, *Oceanospirillales*, *Cellvibrionales*, *Aeromonadales*, *Xanthomonadales*, *Orbales*, *Bacillales* and *Lactobacillales* gave hits in 9% to 67% of the type-material genomes. The rest of orders were mainly negative or just negative, including *Pseudomonadales* and *Legionellales*.

In Table 5, the families pertaining to orders *Enterobacterales* and *Lactobacillales* were analyzed. Among *Enterobacterales*, the families *Morganellaceae*, *Enterobacteriaceae*, *Yersiniaceae* and *Pectobacteriaceae* showed very near to widespread distribution of *cpdB*-like genes, whereas *Erwiniaceae*, *Hafniaceae* and *Budviciaceae* displayed a partial distribution. *Bruguierivoracaceae* fam. was the only one with clearly negative results. Among *Lactobacillales*, all the families exhibited a partial distribution.

In Table 6, selected examples are shown, at the level of species, groups of species, or genus, of clinically relevant bacteria that either contain or do not contain a *cpdB*-like gene. In this case, TblastN analyses were always run without the limit “sequences from type material”; therefore, the results include all the available complete genomes for each species. At this level, 34 species or groups showed a completely or mainly widespread distribution of *cpdB*-like genes, i.e., they were present in 100% or near 100% of the genomes; 10 species showed a partial distribution, with some genomes containing and others not containing *cpdB*-like genes in the same species; and 28 species were negative or mainly negative as they were devoid of *cpdB*-like genes in 100% or near 100% of the genomes. 

Let us discuss now what would be the repercussions of the three kinds of gene distribution found, taking into account that those from *E. coli*, *S. enterica*, *S. agalactiae* and *S. suis* are provirulent in different organisms [9,10,11,14]. Both the presence and the absence of *cpdB*-like genes in the genome can be relevant (although not exclusively, of course) for the virulence degree of the pathogen.

First, for species that did not contain *cpdB*-like genes (i.e., those that in Table 6 are indicated with the N key), it can be safely concluded that these organisms cannot explode the CpdB-like protein-dependent strategy of degrading extracellular cyclic dinucleotides recognized as PAMPs by the infected host [9,10], or of interfering with the complement system [14]. Of course, it is possible that other proteins replace CpdB-like ones. For instance, this occurs in the *Mycobacterium tuberculosis* that is negative for *cpdB*-like genes (Table 6), but expresses a pro-virulent cyclic nucleotide phosphodiesterase, encoded by the *Rv2837c* or *cnpB* gene, which inhibits innate immune cytosolic surveillance [19,71]. Incidentally, this *M. tuberculosisis* protein has been named also CdnP [71], such as the CpdB-like protein of *S. agalactiae* [9], but its encoding gene was not a hit in the TblastN search run with *S. enterica* CpdB (Table 6), as they are very different proteins encoded by different genes. 

Second, concerning species in which *cpdB*-like genes were widespread (i.e., those that in Table 6 are indicated with the W key), they constitute a field where the possible role of these genes in virulence can be explored by constructing gene mutants, and testing them in suitable infection systems in comparison with wildtype bacteria, or by expressing the encoded proteins and studying their enzyme specificity. By extension of what is known about the provirulent role of *cpdB*-like genes, and of CpdB-like enzyme activities, in *E. coli*, *S. enterica*, *S. agalactiae* and *S. suis* [7,8,9,10,11,14], this strategy could be fruitful if applied to other species. For instance, it will be worth exploring genera such as *Bacillus*, *Enterobacter*, *Haemophilus*, *Klebsiella*, *Morganella*, *Pasteurella*, *Proteus*, *Providencia*, *Serratia*, *Shigella* and *Yersinia*, among others, which contain *cpdB*-like genes aligning with high TblastN scores with *S. enterica* CpdB (Table 6). 

Third, particularly interesting are species with a partial distribution of *cpdB*-like genes, indicative that different strains or isolates differ in this concern. This occured very markedly in pathogens like *S. enterica subsp. enterica* ser. Typhimurium, *Streptococcus dysgalactiae* and *Vibrio cholerae*, to mention those that gave higher TblastN scores for alignment with *S. enterica* CpdB (Table 6). In this case, one should consider whether the presence or absence of a *cpdB*-like gene could modulate the virulence of pathogen strains or isolates.

Another interesting observation from Table 6 is that species of the same genus may differ drastically in the content of *cpdB*-like genes. This was the case for genus *Streptococcus*, since all the genomes of *S. agalactiae*, *Streptococcus sanguinis*, *S. suis* (with one exception) and *S. thermophilus* contained a *cpdB*-like gene, but those of *Streptococcus mitis*, *Streptococcus mutans*, *Streptococcus pneumoniae* and *Streptococcus pyogenes* did not, and those of *S. dysgalactiae* and *Streptococcus parasuis* showed a partial distribution. This was confirmed by repeating the TblastN searches using *S. suis* SntA as the query: scores higher than those shown in Table 6 (*S. enterica* CpdB as the query) were obtained, but the distribution of *sntA*-like genes was the same as in Table 6 for every *Streptococcus* species. Another example worthy of comment are the TblastN results with genus *Salmonella*, much more homogeneous in their content of *cpdB*-like genes, which were widespread in *S. bongori* and in *S. enterica* subspecies *arizonae*, *diarizonae*, *houtenau, salamae VII*, and *enterica* serovar Typhi, while it was markedly partial in serovar Typhimurium. Concerning genus *Escherichia*, the presence of *cpdB*-like genes was almost constant, and only a very minor proportion of *E. coli* genomes (0.3%) lack them.

### 2.5. Anecdotal Findings of cpdB-like Genes Outside Eubacteria Chromosomal Genomes

Incidentally, besides the findings summarized in Table 2, Table 3, Table 4, Table 5 and Table 6 for chromosomal genomes, we also observed the presence of *cpdB*-like genes in some unexpected genomic locations, including sequences from: plasmids, *Viruses* (NCBI:txid10239) and *Eukaryota* (NCBI:txid2759). To analyze this as deeply as possible, different ad hoc TblastN searches were ran in the NCBI Nucleotide (nr/nt) database with *S. enterica* CpdB as the query, as described below. 

Within the superkingdom *Archaea*, the TblastN run without any limits, other than the taxonomical one, gave no hits with score > 150. 

Within the superkingdom *Bacteria*, applying the Entrez query “plasmid[Title]”, 41 plasmid sequences containing *cpdB*-like genes with scores ranging 1303–169 were recovered (Table S1). Seven of these hits showed TblastN scores > 1000, with 100% query coverage and >88% identity, and pertained to bacterial species *Salmonella* sp., *Klebsiella pneumoniae*, and *E. coli*. Hits with lower scores corresponded to many different bacteria. The finding of *cpdB*-like genes in plasmids is theoretically in agreement with the protective character of CpdB-like enzymes against innate immune responses of the host [72].

Within the superkingdom *Viruses*, the TblastN run without any limits, other than the taxonomical one, gave two hits (Table S2), one of them with a score of 1062 corresponding to a *cpdB*-like gene of an unclassified bacteriophage of family *Myoviridae* [73,74]. 

Within the superkingdom *Eukaryota*, the TblastN run without any limits, other than the taxonomical one, gave 4 hits (Table S3). Three of them, with score 1185, corresponded to the genome of *Digitaria exilis*, a nutritious cereal known as white fonio that constitutes a vital crop of West Africa [75]. The fourth one, with score 457, corresponded to the genome of *Leishmania major*, a protozoan parasite with the ability to invade macrophages and that causes cutaneous leishmaniasis [76,77].

The presence of *cpdB*-like genes in plasmids, viruses and, particularly, in a higher plant or in a parasitic protozoan is intriguing. One wonders, for instance, whether CpdB-like proteins could have in *Leishmania* the same protective effect versus the immune system of the infected host as they display in *Bacteria*. 

## 3. Materials and Methods 

### 3.1. Cloning, Expression and Purification of Recombinant CpdB

Genomic DNA of *S. enterica subsp. enterica* serovar Typhimurium strain LT2 was acquired from the Colección Española de Cultivos Tipo (CECT, Valencia, Spain). The coding sequence of the mature CpdB protein without signal sequence (GenBank accession number NC_003197.2:c4639575-4641461) was amplified with primers CACTGGGGATCCGCCACCGTCGATCTCCGTATCATGG (forward) and CTGCACGAATTCTTACTTGCTTAAATCCACCTG (reverse), containing, respectively, BamHI and EcoRI sites (underlined). The amplicon was expected to contain the desired coding sequence flanked by those restriction sites. It was obtained with the Advantage cDNA polymerase mix (Clontech), so it contained 3′ A extensions that allowed for T4 DNA ligation to the 3′ T extensions of the pGEM-T Easy vector (Promega). Transformation of competent JM109 cells (Promega) yielded white colonies from where plasmids were obtained (High Pure Plasmid Isolation Kit, Roche). After identification of the correct construct by sequencing, it was cut with BamHI and EcoRI, and the passenger was inserted into the corresponding sites of the pGEX-6P-3 vector in frame with the PreScission protease cut sequence and the glutathione-S-transferase (GST) label. The resulting construct (pGEX-6P-3-S.enter_cpdB) was analyzed by double-strand sequencing (Servicio de Genómica, Instituto de Investigaciones Biomédicas Alberto Sols, Consejo Superior de Investigaciones Científicas, Universidad Autónoma, Madrid). The sequence of the insert matched exactly the genomic coding sequence. 

The expression and purification of the recombinant CpdB was performed as described [7]. In brief: BL-21 cells were transformed with pGEX-6P-3-S.enter_cpdB under ampicillin selection; transformed cells were cultured in suspension, induced by IPTG and collected by centrifugation. After IPTG induction of the *tac* promoter of the vector, the supernatant of the BL-21 cell lysate was used for purification of the GST fusion protein by affinity chromatography on GSH-Sepharose (GE Healthcare Life Sciences) followed by separation from the GST label by specific proteolysis with the PreScission protease (GE Healthcare Life Sciences). This yielded mature CpdB with a GPLGS N-terminal extension, with a purity of 80–85% estimated by SDS gel electrophoresis and image analysis [78].

### 3.2. Enzymatic Assays

All the reactions assayed involved the phosphohydrolysis of monoester, diester or anhydride phosphate linkages. Enzyme incubations were carried out in mixtures containing 50 mM Tris-HCl, pH 7.5 at 37 °C, 2 mM MnCl_2_, 0.1 mg mL^−1^ bovine serum albumin, diverse concentrations of substrate and recombinant enzyme. All the incubations were carried out at 37 °C, under conditions of linearity with respect to incubation length and amount of enzyme. Enzyme-less and/or substrate-less controls were run and subtracted from results of full reaction mixtures. In assays with nucleoside-mono-, di- and triphosphates, and 4-NPhP, the amount of phosphate liberated as a product was measured colorimetrically. For assays with cyclic mononucleotides, diadenosine-oligophosphates, NDP-sugars, CDP-choline, and bis-4NPhP as substrates, an excess of alkaline phosphatase was included in the reaction mixture to liberate phosphate from the reaction products. The colorimetric assay of inorganic phosphate is described elsewhere [7]. The hydrolytic reactions of linear or cyclic oligonucleotides were studied by HPLC monitored at 260 nm, and reaction products separated from substrates were quantitated, as described below. 

The hydrolysis of pApA, c-di-AMP, c-tetra-AMP and c-hexa-AMP was assayed measuring the accumulation of 5′-AMP (respectively, 2 mol, 2 mol, 4 mol or 6 mol per mole of substrate), except for the hydrolysis of c-hexa-AMP by *S. enterica* CpdB, which was assayed measuring substrate consumption. This avoided complications due to the formation of linear oligonucleotides during the reaction progress towards the formation of 5′-AMP. This is in contrast with the behavior of *S. suis* SntA that gave 5′-AMP as the only detectable product, indicating that linear oligonucleotide intermediates were rapidly hydrolyzed. The hydrolysis of pGpG was assayed measuring the accumulation of GpG, 5′-GMP and guanosine. The hydrolysis of 3′,3′-cGAMP was assayed measuring the accumulation of adenosine and guanosine, indicating that 3′-nucleotide products were rapidly dephosphorylated. The very slow hydrolysis of 2′,3′-cGAMP was assayed by the formation of a not well characterized product tentatively identified as G2′p5′A.

Saturation kinetics were studied by measuring initial rates at diverse substrate concentrations. Kinetic parameters *k*_cat_ and *K*_M_ were estimated by nonlinear regression as described [10]. The catalytic efficiency equals the quotient *k*_cat_/*K*_M_, but when saturation plots could not be obtained, this parameter was derived from initial rate data obtained at substrate concentrations much lower than the *K*_M_, i.e., when the initial rate is practically proportional to substrate concentration and most part of the enzyme is in free form. Under these conditions *k*_cat_/*K*_M_ = *v*/([E][S]), [E] being the total concentration of enzyme [79].

### 3.3. HPLC Methods

The HPLC analyses were run on a Tracer Excel 120 column (150 mm × 4 mm) protected by a pre-column (10 mm × 4 mm) of the same material (octadecylsilica; Teknokroma, San Cugat del Vallés, Barcelona). An HP1100 system was used with a diode array detector adjusted to measure *A*_260_. Samples of 20 µL were injected and the elution was performed at 1 mL/min with four different buffers: A, 5 mM Na-phosphate, pH 7.0, 5 mM tetrabutylammonium, 20% methanol (by vol.); B, 100 mM Na-phosphate, pH 7.0, 5 mM tetrabutylammonium, 20% methanol; C, 10 mM Na-phosphate pH 7.0; D, 10 mM Na-phosphate pH 7.0; 50% methanol. The buffers used and the gradient method applied depended on the enzymatic reaction being studied, as indicated below. 

To analyze the hydrolysis of pApA, the initial mobile phase was 80% A, 20% B, and a linear gradient was applied up to 100% B in 10 min.

To analyze the hydrolysis of pGpG, the initial mobile phase was 100% A, and a linear gradient was applied up to 40% A and 60% B in 10 min, followed by another linear gradient up to 30% A and 70% B in 5 min.

To analyze the hydrolysis of c-di-AMP, the initial mobile phase was 100% A, and a linear gradient was applied up to 40% A and 60% B in 5 min, followed by another linear gradient up to 30% A and 70% B in 10 min.

To analyze the hydrolysis of c-di-GMP, the initial mobile phase was 80% A, 20% B, and a linear gradient was applied up to 40% A and 60% B in 10 min.

To analyze the hydrolysis of c-tetra-AMP and c-hexa-AMP, the initial mobile phase was 100% C, and a linear gradient was applied up to 100% D in 10 min.

To analyze the hydrolysis of 2′,3′-cGAMP and 3′,3′-cGAMP, the initial mobile phase was 100% A, and a linear gradient was applied up to 50% A and 50% B in 4 min, followed by another linear gradient up to 100% B in 1 min and isocratic elution with 100%B for 4 min.

### 3.4. Analyses of the Genomic Distribution of cpdB-like Genes

To perform a systematic study of the distribution of *cpdB*-like genes in the Eubacteria superkingdom, we chose to use the protein sequence of *S. enterica* CpdB as query (GenBank accession number P26265), and searched for genomic sequences that, after translation, give significant alignments. The TblastN tool of NCBI was used to this end [64,65]. In the absence of any limits imposed on the search, the complexity of the results would be overwhelming due to the large numbers of microbial genomes currently stored in GenBank for a single species. For instance, a TblastN run with query P26265 against complete genomes of *S. suis* (158 available on 14 December 2022), without any other limit imposed than the organism Taxonomy ID, hits on 127 sequences with scores 409–549 and very significant E values of 10^−144^ and lower. The abundance of sequences for a single species precludes obtaining an unbiased panorama of the genomic distribution in Eubacteria, particularly because species with many genome sequences would be overrepresented in the final panorama. To avoid this bias, and to obtain results in which each species is represented by a single or a small number of genomes, the ability to limit the search to “sequences from type material” was activated in the microbial TblastN launch page [68]. In addition, the Genome database contains many plasmid sequences that count as complete genomes of the host bacterial species. To eliminate these “false” genomes from the TblastN results, the Entrez query “NOT plasmid[Title]” was added to the search. Further to the above strategy, to compute the results obtained in each taxonomy group by alignment to P26265, we imposed that the alignment score should be >150 and the query coverage >70%.

In cases that the above search strategy gave 0-2 hits, and in other selected cases, the search was repeated after removing the limit “sequences from type material” (Table 2, Table 3, Table 4 and Table 5). This was also done for analyses of individual species, genus of specific groups (Table 6). In special cases (Tables S1–S3), the TblastN search was run in the NCBI Nucleotide database (nr/nt).

## 4. Conclusions

*S. enterica* CpdB is a phosphohydrolase of broad specificity with a remarkable selectivity for some substrates versus others. Cyclic and linear dinucleotides are hydrolyzed with efficiencies of 10^4^ M^−1^s^−1^ to 10^7^ M^−1^s^−1^, which makes CpdB capable of acting on these bacterial regulators in the extracytoplasmic media of bacteria, and in the context of host-pathogen interaction. 

*S. enterica* CpdB and *S. suis* SntA hydrolyze, albeit with lower efficiencies, the cyclic oligoadenylates c-tetra-AMP and c-hexa-AMP, and so these enzymes add to the shortlist of phosphodiesterases able to degrade these novel bacterial second messengers. 

In the genomes of superkingdom *Bacteria*, *cpdB*-like genes are far from ubiquitous, as they are present in some phyla but not in others. 

Within phyla containing *cpdB*-like genes, their distribution is not homogeneous, since at any taxonomical level above species there are few taxa containing a *cpdB*-like gene in all the sequenced genomes. 

At the level of species, the distribution is more homogeneous, as out of 77 taxa investigated, 38 show a widespread or near widespread distribution of *cpdB*-like genes, 11 show a partial distribution, and 28 do not contain *cpdB*-like genes.

Species that do not contain *cpdB*-like genes cannot explode the CpdB-like protein-dependent strategy of degrading extracellular cyclic dinucleotides recognized as PAMPs by the infected host.

Species in which *cpdB*-like genes are widespread constitute a field where the possible role of these genes in virulence can be explored by creating gene mutants and studying the enzyme specificity of the CpdB-like protein.

In species with a partial distribution of *cpdB*-like genes, the presence or absence of a *cpdB*-like gene could modulate the virulence of pathogen strains or isolates.

## Figures and Tables

**Table 1:** Kinetic parameters of *S. enterica* CpdB. New data for *S. suis* SntA are shown in the lower part of the table. Data are mean values ± standard deviations of three experiments.

	*k*_cat_^1^	*K*_m_^1^	*k*_cat_/*K*_m_^1^
	(s^−1^)	(µM)	(M^−1^s^−1^)
*S. enterica* CpdB			
3′-GMP	219.07 ± 48.74	4.66 ± 1.34	5.23 × 10^7^
3′-UMP	154.93 ± 13.31	4.54 ± 0.53	3.42 × 10^7^
2′,3′-cCMP	146.18 ± 9.39	4.52 ± 0.69	3.32 × 10^7^
3′-AMP	171.85 ± 41.34	6.53 ± 1.16	2.76 × 10^7^
2′,3′-cGMP	120.04 ± 4.74	7.52 ± 1.46	1.63 × 10^7^
2′,3′-cAMP	209.47 ± 16.94	14.41 ± 3.68	1.53 × 10^7^
2′,3′-cUMP	324.03 ± 21.76	22.65 ± 2.56	1.44 × 10^7^
pApA	2.85 ± 1.04	0.22 ± 0.12	1.39 × 10^7^
bis-4NPhP	250.22 ± 9.82	33.74 ± 4.77	7.53 × 10^6^
pGpG	2.27 ± 1.95	3.07 ± 3.67	9.88 × 10^5^
4-NPhP	24.89 ± 2.54	92.94 ± 44.66	3.01 × 10^5^
3′,3′-cGAMP	0.30 ± 0.03	2.13 ± 0.32	1.97 × 10^5^
ATP	23.06 ± 2.74	137.42 ± 59.23	1.82 × 10^5^
ADP	2.83 ± 0.19	28.09 ± 1.82	1.01 × 10^5^
Ap4A	2.64 ± 0.24	49.21 ± 12.46	5.50 × 10^4^
c-di-AMP	0.31 ± 0.06	8.89 ± 2.31	3.55 × 10^4^
Ap3A	2.10 ± 0.17	69.09 ± 19.31	3.16 × 10^4^
ADP-ribose	2.40 ± 0.36	99.61 ± 15.81	2.44 × 10^4^
c-di-GMP	0.09 ± 0.01	4.12 ± 0.98	2.24 × 10^4^
CDP-choline	2.84 ± 0.30	191.82 ± 19.71	1.49 × 10^4^
c-tetra-AMP	0.042 ± 0.001	18.44 ± 4.95	2.37 × 10^3^
3′,5′-cAMP	0.67 ± 0.32	2444.10 ± 1337.05	2.94 × 10^2^
c-hexa-AMP	0.006 ± 0.001	24.24 ± 7.60	2.38 × 10^2^
ADP-glucose	0.06 ± 0.02	299.39 ± 105.92	2.13 × 10^2^
UDP-glucose	0.12 ± 0.02	851.05 ± 216.31	1.46 × 10^2^
2′,3′-cGAMP	Nd	Nd	9.06 × 10^1^
5′-AMP ^2^	Nd	Nd	Nd
2′-AMP ^2^	Nd	Nd	Nd
*S. suis* SntA			
c-tetra-AMP	1.13 ± 0.16	262.5 ± 62.14	4.38 × 10^3^
c-hexa-AMP	0.11 ± 0.01	2.63 ± 0.88	4.57 × 10^4^

^1^*k*_cat_ and *K*_M_ were calculated from saturation curves obtained at different concentrations of substrate; the catalytic efficiencies were calculated by dividing *k*_cat_/*K*_M_ or, when these parameters were not available (2′,3′-cGAMP), by the procedure described in Section 3.2. ^2^ In assays at a fixed 500 µM concentration, the activities on 5′-AMP and 2′-AMP represented less than 0.0006% of the activity on 3′-GMP and less than 2% of the activity on 3′,5′-cAMP. Nd: not determined.

**Table 2:** Distribution of *cpdB*-like genes in different phyla or groups of phyla of the superkingdom *Bacteria*, as obtained by TblastN analysis using *S. enterica* CpdB as the query ^1^.

Phylum or Group of Phyla	Taxonomy ID	Genome Hits/Total ^2^		Hit Score Range		Key ^3^
*Proteobacteria*	1224	530/1756		1307–181		P
*Firmicutes*	1239	248 ^4^/644		681–155 ^4^		P
*Deinococcus-Thermus*	1297	23/45		600–557		P
*Spirochaetes*	203691	12/57		554–160		P
*Thermotogae*	200918	9/23		376–167		P
*Actinobacteria*	201174	116 ^4^/668		334–192 ^4^		P
*FCB group*	1783270	32/291		244–152		P
*Coprothermobacterota*	2138240	1/1	(1/1)	258	(258)	Wm
*Calditrichaeota*	1930617	1/1	(1/1)	167	(167)	Wm
*PVC group*	1783257	2/89	(2/393)	239–221	(293–221)	Nm
*Fusobacteria*	32066	1/14	(3/74)	193	(193–154)	Nm
*Tenericutes*	544448	1/121	(2/499)	166	(166–162)	Nm
*Cyanobacteria*	1117	0/28	(2/221)	–	(238–237)	Nm
*Armatimonadetes*	67819	0/1	(1/5)	–	(166)	Nm
*Acidobacteria*	57723	0/18	(1/47)	–	(206)	Nm
*Chloroflexi*	200795	0/17	(0/51)	–	(–)	N
*Aquificae*	200783	0/10	(0/16)	–	(–)	N
*Deferribacteres*	200930	0/6	(0/8)	–	(–)	N
*Thermodesulfobacteria*	200940	0/6	(0/7)	–	(–)	N
*Synergistetes*	508458	0/5	(0/8)	–	(–)	N
*Nitrospirae*	40117	0/3	(0/13)	–	(–)	N
*Dictyoglomi*	68297	0/2	(0/2)	–	(–)	N
*Elusimicrobia*	74152	0/2	(0/5)	–	(–)	N
*Atribacterota*	67818	0/1	(0/1)	–	(–)	N
*Caldiserica/Cryosericota group*	2498710	0/1	(0/1)	–	(–)	N
*Chrysiogenetes*	200938	0/1	(0/2)	–	(–)	N
*Nitrospinae/Tectomicrobia group*	1802340	0/1	(0/1)	–	(–)	N

^1^ Those phyla without any fully sequenced genome of type material were not included in the table. ^2^ A “Genome hit” corresponds to a TblastN alignment with score > 150 and query coverage > 70% in the Complete Genomes database (limited to “sequences from type material”, records without “plasmid” in the title, and the indicated taxonomy ID). “Total” refers to the number of sequences in the database with the same limits. Data in parenthesis were obtained by removing the search limit “sequences from type material”. Access on 13–23 December 2022. The numbers vary slightly depending on accession date. ^3^ The key indicates, in a somewhat subjective manner, the presence of *cpdB*-like genes in the bacterial taxa: W, widespread; P, partial; N, negative; m, mainly (as a modifier of W or N; indicating that there are a few exceptions and/or there is only a single genome available). ^4^ In these cases, one hit with score 151 (in Actinobacteria) or 152 (in Firmicutes) was removed as it showed higher BlastP alignment scores with UshA than with CpdB (see the text).

**Table 3:** Distribution of *cpdB*-like genes in different classes of phyla *Proteobacteria* and *Firmicutes*, as obtained by TblastN analysis using *S. enterica* CpdB as the query.

Class	Taxonomy ID	Genome Hits/Total ^1^		Hit Score Range		Key ^2^
Phylum *Proteobacteria*						
*Gammaproteobacteria*	1236	345/813		1307–362		P
*Betaproteobacteria*	28216	68/333		776–395		P
*Alphaproteobacteria*	28211	107/409		647–245		P
*Delta/epsilon subdivisions*	68525	10/190		640–181		P
*Oligoflexia*	1553900	0/4	(1/17)	–	(672)	Nm
*Acidithiobacillia*	1807140	0/4	(0/17)	–	(–)	N
*Zetaproteobacteria*	580370	0/2	(0/2)	–	(–)	N
*Hydrogenophilalia*	2008785	0/1	(0/1)	–	(–)	N
Phylum *Firmicutes*						
*Bacilli*	91061	194/384		681–155		P
*Clostridia*	186801	48 ^3^/215		509–155 ^3^		P
*Erysipelotrichia*	526524	4/15		176–156		P
*Limnochordia*	1676648	1/1	(1/1)	420	(420)	Wm
*Negativicutes*	909932	1/16	(2/41)	240	(240–201)	Nm
*Tissierellia*	1737404	0/12	(0/26)	–	(–)	N

^1,2,3^ See footers 2, 3 and 4 of Table 2, respectively.

**Table 4:** Distribution of *cpdB*-like genes in different orders of classes *Gammaproteobacteria* and *Bacilli*, as obtained by TblastN analysis using *S. enterica* CpdB as the query.

Order	Taxonomy ID	Genome Hits/Total ^1^		Hit Score Range		Key ^2^
Class *Gammaproteobacteria*						
*Pasteurellales*	135625	31/31		875–813		W
*Enterobacterales*	91347	166/181		1307–547		Wm
*Moraxellales*	2887326	3/32		1031–455		P
*Vibrionales*	135623	70/153		930–376		P
*Alteromonadales*	135622	39/93		912–362		P
*Oceanospirillales*	135619	13/47		867–560		P
*Cellvibrionales*	1706369	3/18		656–640		P
*Aeromonadales*	135624	6/10		595–554		P
*Xanthomonadales*	135614	10/45		452–375		P
*Orbales*	1240482	2/3	(5/6)	876–796	(876–796)	P
*Chromatiales*	135613	1/29	(1/38)	591	(591)	Nm
*Thiotrichales*	72273	1/33	(2/205)	460	(460–459)	Nm
*Pseudomonadales*	72274	0/85	(1/1348)	—	(664)	Nm
*Legionellales*	118969	0/28	(0/176)	—	(–)	N
*Methylococcales*	135618	0/8	(0/30)	—	(–)	N
*Kangiellales*	2887327	0/4	(0/4)	—	(–)	N
*Acidiferrobacterales*	1692040	0/2	(0/4)	—	(–)	N
*Nevskiales*	1775403	0/2	(0/4)	—	(–)	N
*Cardiobacteriales*	135615	0/1	(0/4)	—	(–)	N
*Immundisolibacterales*	1934945	0/1	(0/1)	—	(–)	N
Class *Bacilli*						
*Bacillales*	1385	125/218		681–155		P
*Lactobacillales*	186826	69/166		556–156		P

^1,2^ See footers 2 and 3 of Table 2, respectively.

**Table 5:** Distribution of *cpdB*-like genes in different families of orders *Enterobacterales* and *Lactobacillales*, as obtained by TblastN analysis using *S. enterica* CpdB as the query.

Family	Taxonomy ID	Genome Hits/Total ^1^		Hit Score Range		Key ^2^
Order *Enterobacterales*						
*Morganellaceae*	1903414	9/9	(260/267)	976–547	(982–547)	Wm
*Enterobacteriaceae*	543	81/83		1307–826		Wm
*Yersiniaceae*	1903411	50/52	(329/337)	1087–1045	(1097–754)	Wm
*Pectobacteriaceae*	1903410	14/15	(144/149)	1074–1060	(1075–734)	Wm
*Erwiniaceae*	1903409	10/13	(102/174)	1075–662	(1078–662)	P
*Hafniaceae*	1903412	1/3	(10/42)	1046	(1050–1042)	P
*Budviciaceae*	1903416	0/3	(2/6)	—	(904–904)	P
*Bruguierivoracaceae*	2812006	0/1	(0/9)	—	(–)	N
Order *Lactobacillales*						
*Streptococcaceae*	1300	21/64		556–170		P
*Lactobacillaceae*	33958	27/61		221–156		P
*Enterococcaceae*	81852	13/17		227–179		P
*Carnobacteriaceae*	186828	4/8		208–180		P
*Aerococcaceae*	186827	2/9	(5/17)	194	(196–194)	P

^1,2^ See footers 2 and 3 of Table 2, respectively.

**Table 6:** Selected pathogens that contain or do not contain *cpdB*-like genes, as obtained by TblastN analysis using *S. enterica* CpdB as the query without limiting to “sequences from type material”.

Specific Name	Taxonomy ID	Group in Table 2, Table 3, Table 4 and Table 5	Genome Hits/Total ^1^	Hit Score Range	Key ^2^
*Aeromonas hydrophila*	644	*Aeromonadales*	56/56	597–445	W
*Bacillus anthracis*	1392	*Bacillales*	107/107	556–541	W
*Bacillus cereus*	1396	*Bacillales*	122/122	558–449	W
*Bacillus subtilis*	1423	*Bacillales*	234/234	557–537	W
*Bacillus subtilis group* (excluding *B. subtilis*)	653685	*Bacillales*	331/332	563–311	Wm
*Citrobacter* (genus)	544	*Enterobacteriaceae*	206/206	1248–1187	W
*Clostridium perfringens*	1502	*Clostridia*	58/60	175–165	Wm
*Clostridium tetani*	1513	*Clostridia*	3/3	157–155	W
*Enterobacter cloacae complex*	354276	*Enterobacteriaceae*	481/483	1207–535	Wm
*Escherichia* (genus) (excluding *E. coli)*	561	*Enterobacteriaceae*	113/113	1203–523	W
*Escherichia coli*	562	*Enterobacteriaceae*	3014/3023	1200–240	Wm
*Haemophilus influenzae*	727	*Pasteurellales*	92/92	865–850	W
*Haemophilus parainfluenzae*	729	*Pasteurellales*	16/16	870–860	W
*Hafnia alvei*	569	*Hafniaceae*	6/6	1050–1042	W
*Helicobacter pylori*	210	*Delta/epsilon subdiv.*	252/254	209–177	Wm
*Klebsiella* (genus)	570	*Enterobacteriaceae*	2191/2196	1204–318	Wm
*Kluyvera ascorbata*	51288	*Enterobacteriaceae*	1/1	1208	Wm
*Morganella morganii*	582	*Morganellaceae*	50/50	981–547	W
*Pasteurella multocida*	747	*Pasteurellales*	133/134	843–469	Wm
*Plesiomonas shigelloides*	703	*Enterobacteriaceae*	5/6	1016–1015	Wm
*Proteus mirabilis*	584	*Morganellaceae*	98/98	936–932	W
*Proteus vulgaris*	585	*Morganellaceae*	11/11	937–927	W
*Providencia stuartii*	588	*Morganellaceae*	14/15	976–946	Wm
*Salmonella bongori*	54736	*Enterobacteriaceae*	12/12	1273–1264	W
*S. enterica subsp. arizonae*	59203	*Enterobacteriaceae*	8/8	1291–1274	W
*S. enterica subsp. diarizonae*	59204	*Enterobacteriaceae*	12/12	1291–1290	W
*S. enterica subsp. enterica* serovar Typhi	90370	*Enterobacteriaceae*	110/110	1296–1295	W
*S. enterica subsp. houtenae*	59205	*Enterobacteriaceae*	5/5	1290–1273	W
*S. enterica subsp. salamae*	59202	*Enterobacteriaceae*	14/14	1295–729	W
*S. enterica subsp. VII*	59208	*Enterobacteriaceae*	2/2	1278–1274	W
*Serratia liquefaciens*	614	*Yersiniaceae*	8/8	1081–1075	W
*Serratia marcescens*	615	*Yersiniaceae*	138/139	1086–996	Wm
*Shigella* (genus)	620	*Enterobacteriaceae*	176/176	1197–650	W
*Streptococcus agalactiae*	1311	*Streptococcaceae*	87/87	545–248	W
*Streptococcus sanguinis*	1305	*Streptococcaceae*	8/8	547–540	W
*Streptococcus suis*	1307	*Streptococcaceae*	115/116	549–409	Wm
*Streptococcus thermophilus*	1308	*Streptococcaceae*	84/84	504–152	W
*Yersinia* (genus)	629	*Yersiniaceae*	171/171	1097–754	W
*Clostridium botulinum*	1491	*Clostridia*	10/65	167–151	P
*Enterococcus avium*	33945	*Enterococcaceae*	2/3	208–207	P
*Enterococcus faecalis*	1351	*Enterococcaceae*	91/97	216–208	P
*Enterococcus faecium*	1352	*Enterococcaceae*	34/305	196–196	P
*Salmonella enterica*	28901	*Enterobacteriaceae*	1613/1750	1307–224	P
*S. enterica subsp. enterica* ser. Typhimurium	90371	*Enterobacteriaceae*	204/335	1307–1043	P
*Staphylococcus epidermidis*	1282	*Bacillales*	116/125	137–130	P
*Staphylococcus saprophyticus*	29385	*Bacillales*	4/16	169–167	P
*Streptococcus dysgalactiae*	1334	*Streptococcaceae*	13/26	543–158	P
*Streptococcus parasuis*	1501662	*Streptococcaceae*	3/6	538–536	P
*Vibrio cholerae*	666	*Vibrionales*	110/217	920–365	P
*Acinetobacter calcoaceticus/baumannii complex*	909	*Moraxellales*	0/590	–	N
*Aerococcus urinae*	1376	*Aerococcaceae*	0/3	–	N
*Borrelia burgdorferi*	139	*Spirochaetes*	0/11	–	N
*Brucella* (genus)	234	*Alphaproteobacteria*	0/322	–	N
*Campylobacter jejuni*	197	*Delta/epsilon subdiv.*	0/321	–	N
*Chlamydia* (genus)	810	*PVC group*	0/188	–	N
*Clostridiodis difficile*	1496	*Clostridia*	0/118	–	N
*Corynebacterium diphtheriae*	1717	*Actinobacteria*	0/71	–	N
*Coxiella burnetii*	777	*Legionellales*	0/16	–	N
*Francisella tularensis*	263	*Thiotrichales*	0/62	–	N
*Legionella pneumophila*	446	*Legionellales*	0/111	–	N
*Leptospira* (genus)	171	*Spirochaetes*	0/160	–	N
*Listeria monocytogenes*	1639	*Bacillales*	0/282	–	N
*Moraxella catarrhalis*	480	*Moraxellales*	0/12	–	N
*Mycobacterium* (genus)	1763	*Actinobacteria*	0/510	–	N
*Mycoplasma* (genus)	2093	*Tenericutes*	0/118	–	N
*Neisseria* (genus)	487	*Betaproteobacteria*	0/306	–	N
*Pseudomonas aeruginosa*	287	*Pseudomonadales*	0/509	–	N
*Pseudomonas fluorescens gr.*	136843	*Pseudomonadales*	0/107	–	N
*Rickettsia rickettsii*	783	*Alphaproteobacteria*	0/14	–	N
*Staphylococcus aureus*	1280	*Bacillales*	0/1105	–	N
*Staphylococcus warnerii*	1292	*Bacillales*	0/10	–	N
*Stenotrophomonas maltophilia group*	995085	*Xanthomonadales*	0/61	–	N
*Streptococcus mitis*	28037	*Streptococcaceae*	0/10	–	N
*Streptococcus mutans*	1309	*Streptococcaceae*	0/21	–	N
*Streptococcus pneumoniae*	1313	*Streptococcaceae*	0/143	–	N
*Streptococcus pyogenes*	1314	*Streptococcaceae*	0/253	–	N
*Treponema pallidum*	160	*Spirochaetes*	0/9	–	N

^1^ In this table, a “Genome hit” corresponds to a TblastN alignment with a score > 150 and a query cover > 70% in the Complete Genomes database (limited to records of the indicated taxonomy ID, without “plasmid” in the title). “Total” refers to the number of sequences in the database with the same limits. ^2^ See footer of Table 2. The results are ordered by distribution key (W or Wm), P, (Nm or N), and alphabetically within each key.

## Data Availability

The supporting raw data may be requested from the corresponding author without reservation.

## Supplementary Materials

The following supporting information can be downloaded at: https://www.mdpi.com/article/10.3390/ijms24044150/s1.

## Abbreviations

2′,3′-cAMP	2′,3′-cyclic monoadenylate
2′,3′-cCMP	2′,3′-cyclic monocitidylate
2′,3′-cGAMP	2′,3′-cyclic GMP-AMP
2′,3′-cGMP	2′,3′-cyclic monoguanylate
2′,3′-cNMP	2′,3′-cyclic mononucleoside monophosphate
2′,3′-cUMP	2′,3′-cyclic monouridylate
3′,3′-cGAMP	3′,3′-cyclic GMP-AMP
3′,5′-cAMP	3′,5′-cyclic monoadenylate
4-NPhP	4-nitrophenylphosphate
ADP-glucose	adenosine 5′-diphosphoglucose
ADP-ribose	adenosine 5′-diphosphoribose
Ap3A	diadenosine triphosphate
Ap4A	diadenosine tetraphosphate
bis-4NPhP	bis-4-nitrophenylphosphate
c-di-AMP	cyclic-3′,5′-diadenylate
c-di-GMP	cyclic-3′,5′-diguanylate
c-di-NMP	cyclic-3′,5′-dinucleotide
c-hexa-AMP	cyclic-3′,5′-hexanucleotide
c-tetra-AMP	cyclic-3′,5′-tetranucleotide
CDP-choline	cytidine 5′-diphosphocholine
G2′p5′A	guanosine-2′,5′-AMP
GST	glutathione -S-transferase
HPLC	high performance liquid chromatography
NDP-hexose	nucleoside 5′-diphosphohexose
PAMP	pathogen-associated molecular pattern
pApA	linear-3′,5′-diadenylate
pGpG	linear-3′,5′-diaguanylate
pNpN	linear-3′,5′-dinucleotide
RNase	ribonuclease
STING	stimulator of interferon genes
UDP-glucose	uridine 5′-diphosphoglucose
UDP-sugar	uridine 5′-diphosphosugar
